# Deprivation indices and their association with fragility fractures and bone density: evidence from a large observational cohort

**DOI:** 10.1093/rheumatology/keaf550

**Published:** 2025-10-17

**Authors:** Hamzah Amin, Muhammed Aqib Khan, Marwan Bukhari

**Affiliations:** Lancaster University Medical School, Faculty of Health and Medicine, Lancaster University, Lancaster, UK; Department of Rheumatology, University Hospitals of Morecambe Bay NHS Foundation Trust, Lancaster, UK; Lancaster University Medical School, Faculty of Health and Medicine, Lancaster University, Lancaster, UK; Department of Rheumatology, University Hospitals of Morecambe Bay NHS Foundation Trust, Lancaster, UK

**Keywords:** deprivation, socioeconomic status, fragility fractures, bone density, body composition, IMD, Townsend score

## Abstract

**Objectives:**

Socioeconomic deprivation as a fracture risk factor remains underexplored. We evaluated associations between deprivation indices and bone health outcomes in a UK clinical population.

**Methods:**

A total of 40 951 patients aged ≥50 years underwent DXA scanning between June 2004 and May 2025 in northwest England. Socioeconomic status was assessed using the Index of Multiple Deprivation (IMD) and Townsend Deprivation Score (TDS). Generalised additive models examined associations between deprivation and major osteoporotic fractures (MOFs), hip fractures (HFs), bone density and body composition while adjusting for FRAX risk factors.

**Results:**

Of the 40 951 patients who underwent DXA scanning, 32 324 (79%) were women with mean age 68.2 years and 11 811 MOFs including 2208 hip fractures. After excluding patients with missing deprivation data, 29 693 patients were analysed. The most deprived patients (IMD) had higher odds of MOF (OR 1.07, 95% CI 1.02–1.14) and HF (OR 1.28, 95% CI 1.11–1.46). TDS was also associated with MOF (OR 1.10, 95% CI 1.03–1.18). Both indices were linked to higher osteoporosis odds: TDS showed ORs of 1.45 (95% CI 1.33–1.59) for femoral neck and 1.30 (95% CI 1.19–1.29) for lumbar spine, while IMD showed ORs of 1.34 (95% CI 1.24–1.45) and 1.20 (95% CI 1.13–1.29), respectively. Deprived patients had increased regional body fat: TDS had 0.90% higher femoral fat and 0.84% higher abdominal fat, while IMD showed 0.60% and 0.67% higher fat percentages, respectively.

**Conclusions:**

Socioeconomic deprivation is independently associated with increased fracture odds, osteoporosis and high fragility fracture risk-related body composition.

Rheumatology key messages

- Index of Multiple Deprivation (IMD) is associated with increased odds of hip fracture.
- The Townsend deprivation score and IMD are associated with increased odds of osteoporosis.
- The Townsend deprivation score and IMD are associated with increased regional body fat.

## Introduction

Osteoporosis is a skeletal disorder characterized by bone mineral density (BMD) that is 2.5 standard deviations below the mean of a young healthy reference population [1]. It remains a significant public health concern due to its strong association with fragility fractures [2, 3]. These fractures occur from low-energy trauma that would not ordinarily cause fracture in a healthy young adult [4]. Fragility fractures most commonly involve the hip, vertebrae and wrist and are linked to substantial increases in morbidity, mortality and healthcare costs [5]. In the UK alone, the annual economic burden of osteoporotic fractures has been estimated at £4.6 billion, a figure expected to rise with an ageing population [6].

As osteoporosis is asymptomatic until a fracture occurs, early identification of individuals is conducted via fracture risk tools. Examples of these tools include FRAX and QFracture which estimate 10-year fracture risk using clinical risk factors with or without the inclusion of BMD [7]. While these models have advanced fracture risk algorithms, many individuals who sustain fragility fractures are classified as low or intermediate risk, and a considerable proportion have fractures despite their BMD values being in the non-osteoporotic range [8]. This highlights the limitations of current approaches and the need for additional predictors to enhance risk identification. One potentially important but underexplored risk factor is socioeconomic deprivation.

Socioeconomic status reflects an individual’s access to resources, opportunities and services and is often assessed using indicators such as education, income, occupation, housing quality and healthcare access [9]. A meta-analysis by Valentin *et al.* suggested that individuals of lower socioeconomic status may have up to a 25% increased risk of fragility fractures compared with more affluent individuals, though this association appeared to be mediated by differences in body composition and lifestyle factors [10]. Importantly, the study also noted high heterogeneity across included studies, with differing measures of socioeconomic status and varying adjustment for confounders, limiting the generalizability of their findings [10].

Within the UK there remains a paucity of high-quality data examining the association between deprivation and fracture risk. In the meta-analysis by Valentin *et al.* [10] only one study was included from the UK. This study by Sydall *et al.* using the Hertfordshire Cohort Study and the Registrar General’s Social Class scale, a measure no longer commonly used, found no association between social class and fracture [11]. More recently, Mahmud *et al.* using UK Biobank data and the Townsend Deprivation Score (TDS) found that greater deprivation was associated with poorer bone health and increased fracture odds [12].

Among the various deprivation indices, the Index of Multiple Deprivation (IMD) is widely used in health research [13]. It is a composite, area-based measure derived from seven domains including income, education, employment, health and living environment, reflecting broader community-level deprivation [13]. Despite its widespread use, the relationship between IMD, fracture risk and bone density has not been studied to our knowledge. Furthermore, no studies have directly compared multiple deprivation indices to identify which measure may be most useful in fracture risk tools or for public health planning.

### Aims

The aim of this study is to evaluate and compare the associations between socioeconomic deprivation and bone health using multiple deprivation indices in a large UK clinical cohort. Specifically, we will compare the IMD and the TDS in relation to fragility fractures, bone density and regional body composition.

## Methods

### Study population and data collection

Between June 2004 and May 2025, 46 487 patients were referred for a DXA scan at our centre in northwest England. At the time of each scan, a trained technician conducted a standardised consultation using a structured questionnaire to assess established fracture risk factors including current or previous glucocorticoid use (prednisolone equivalent ≥5 mg daily for ≥3 months), previous fragility fracture, family history of hip fracture, current smoking status, alcohol consumption (≥3 units daily), RA and measuring the patient’s BMI.

Patients were asked to self-report any previous fragility fractures, defined as fractures resulting from the trauma equivalent of a fall from standing height or less. The mechanism and anatomical site of any reported fractures were verified against the patient’s medical history. The majority of patients underwent bilateral hip and lumbar spine (L1–L4) DXA scans using the lunar prodigy between 2004 and 2019 and more recently the iDXA machine from 2019 to the present day. Both machines were cross calibrated for methodological consistency and are quality controlled weekly using scanning phantoms.

Demographic data including age, sex and residential postcode were also available for participants.

### Deprivation index calculation: IMD

IMD scores and deciles were derived from patient postcodes at the lower super output area level using the ‘IMD’ package in R statistical software, which is based on 2019 IMD data. The 2019 IMD data represents the most recent deprivation data published and provides deciles and rankings across seven domains: income, employment, education, health, crime, barriers to housing and services and living environment.

While ideally, we would have used IMD data that was closest to each patient’s scan date, temporal inconsistencies in IMD methodology and component weightings across different years would prevent meaningful analysis [14]. Therefore, we decided that IMD 2019 results be applied consistently across all scan dates for methodological consistency.

### Deprivation index calculation: TDS

TDS values were calculated using the ‘QDiabetes’ package in R, based on 2011 census data linked to patient postcodes. The TDS is a composite measure of material deprivation derived from four census variables: unemployment (percentage of economically active residents aged 16–59/64 who are unemployed), overcrowding (percentage of households with more than one person per room), non-car ownership (percentage of households without access to a car) and non-home ownership (percentage of households not owner-occupied). Given the recency of the 2021 census, the 2021 Townsend scores are not widely available for use in healthcare research with studies still predominantly using the 2011 values, hence the rationale for the inclusion in our study.

### Statistical analysis

Patients aged 50 years and older were included in the analysis. Baseline demographic characteristics and fracture risk factors were first compared between the most deprived (IMD deciles 1–5) and the least deprived (IMD deciles 6–10) groups. For continuous variables, Student’s *t*-test was used, assuming normality based on the large sample size and the Central Limit Theorem. Categorical variables were compared using the *χ*^2^ test.

Deprivation was categorized into binary variables. Participants were classified as ‘most deprived’ or ‘least deprived’ using the IMD, with deciles 1–5 defined as most deprived and 6–10 as least deprived. For TDS, values >0 were classified as most deprived and ≤0 as least deprived, consistent with its national centreing around zero. Binary categorization was chosen to address potential non-linearity and ensure easy interpretation of results, particularly given many outcomes we were investigating. Although IMD could theoretically be modelled as a multilevel factor, this approach was deemed sub-optimal due to the large number of levels and the likelihood of small fracture counts within individual strata, which would compromise statistical power.

Generalised additive models (GAMs) with a binomial distribution were fitted to assess associations between deprivation and major osteoporotic fractures (MOFs), defined as fractures of the spine, femur, humerus, forearm or radius/ulna. Separate models were used for IMD decile and TDS as primary predictors as opposed to including both measures in a single model. All models were adjusted for potential confounders including age at scan (modelled as a smooth term), sex, current smoking status, current excessive alcohol consumption, current steroid therapy, RA, family history of fracture and BMI (modelled as a smooth term) and the left femoral neck *T*-score (modelled as a smooth term). Only the left femoral neck *T*-score was included in the model as this the site is most often affected by OP [15]. Additionally, the inclusion of multiple bone density measures may also lead to collinearity concerns which could cause numerical instability in our coefficient estimates. Models were also constructed to model hip fractures using the same approach.

Additional models examined the association between deprivation and binary osteoporosis status, based on *T*-scores <-2.5 standard deviations at the left femoral neck and lumbar spine as separate outcomes and using the same covariates (excluding the respective *T*-score as an adjustment variable). Further models assessed deprivation in relation to continuous body composition variables, specifically left femoral fat percentage and abdominal fat percentage, again adjusting for the same confounding variables.

### Supplementary analysis

Even though we opted for using a binary IMD and TDS approach in our main analysis we nonetheless conducted a supplementary analysis using the same approach as described above however we included IMD as a multilevel factor with the reference level being IMD decile 1 (most deprived) and modelled TDS as a continuous variable using a smoothing spline with the same outcomes. This analysis is reported in full in the Supplementary Material.

### Ethics statement

Full ethical approval for pseudonymized data extraction in the absence of informed consent was obtained from the regional NHS Research Ethics Committee Northwest Preston (project number 14/NW/1136).

## Results

### Baseline demographics and risk factors summary statistics

A total of 40 951 patients met the eligibility criteria for the study. A total of 11 811 MOFs were reported including 2208 hip fractures. The sample consisted of 32 324 (79%) female patients, with a mean age of 68.23 years (S.D. 9.92). The mean TDS was −1.98 (S.D. 2.38), and the mean IMD decile was 6.11 (S.D. 2.60). A total of ∼11 000 patients were excluded from the primary analysis due to missing TDS or IMD data, resulting in a final sample of 29 693 patients in our regression models. This was primarily attributable to incomplete postcode data in the original dataset, with a smaller proportion resulting from errors encountered when extracting deprivation indices. Across the full (40 951 patients) cohort, the mean femoral neck BMD *T*-score was −1.29 (S.D. 1.16) and the mean BMI was 27.15 kg/m^2^ (S.D. 5.42).

Significant differences in fracture risk factors were observed between patients in the most and least deprived IMD groups. Individuals in the most deprived group (*n* = 10 659) had higher rates of current smoking (16.0% vs 7.5%), higher mean BMI (27.74 vs 26.82 kg/m^2^), a greater prevalence of RA (8.0% vs 6.5%) and lower mean femoral neck BMD *T*-scores (−1.33 vs −1.29), all *P* < 0.001. Additionally, patients in the most deprived groups were more likely to report MOF (31.2% vs 29.9%, *P* = 0.002) and hip fractures (5.9% vs 5.0%, *P* < 0.001). In contrast, those in the least deprived group (*n* = 19 134) were marginally older and more frequently reported a family history of fracture. No significant differences were observed between the groups in the prevalence of steroid therapy use or excess alcohol consumption. Full results are presented in Table 1.

**Table 1.** Baseline demographics and risk factors by deprivation status

Characteristic	Overall	Least deprived	Most deprived	*P*-value^2^
	(*n* = 29 793)^1^	(*n* = 19 134; 64.2%)^1^	(*n* = 10 659; 35.8%)^1^	
Age at scan (years)	67.93 (9.86)	68.28 (9.87)	67.31 (9.80)	<0.001
Gender				0.02
Gender (female)	24 211 (81.3%)	15 624 (81.7%)	8587 (80.6%)	
Gender (male)	5582 (18.7%)	3510 (18.3%)	2072 (19.4%)	
BMI (kg/m^2^)	27.15 (5.42)	26.82 (5.12)	27.74 (5.88)	<0.001
Current steroid therapy	3465 (11.6%)	2270 (11.9%)	1195 (11.2%)	0.092
RA	2095 (7.0%)	1238 (6.5%)	857 (8.0%)	<0.001
Excess alcohol consumption	1758 (5.9%)	1133 (5.9%)	625 (5.9%)	0.8
Current smoking	3.133 (10.5%)	1432 (7.5%)	1701 (16.0%)	<0.001
Family history of fracture	5689 (19.1%)	3772 (19.7%)	1917 (18.0%)	<0.001
Femoral neck BMD *T*-score	−1.30 (1.15)	−1.29 (1.14)	−1.33 (1.17)	0.002
Lumbar spine BMD *T*-score	−0.68 (1.97)	−0.65 (1.96)	−0.72 (1.98)	0.002
Major osteoporotic fracture	9053 (30.4%)	5726 (29.9%)	3327 (31.2%)	0.021
Hip fracture	1590 (5.3%)	959 (5.0%)	631 (5.9%)	<0.001
^1^Mean (S.D.); *n* (%)				
^2^Wilcoxon rank sum test; Pearson’s *χ*^2^ test				

Comparison of baseline characteristics between most deprived (IMD deciles 1–5) and least deprived (IMD deciles 6–10) patients undergoing DXA scanning. Most deprived patients demonstrated significantly higher rates of current smoking (16.0% vs 7.5%), elevated BMI (27.74 vs 26.82 kg/m^2^), increased RA prevalence (8.0% vs 6.5%) and lower femoral neck BMD *T*-scores (−1.33 vs −1.29). Major osteoporotic fractures and hip fractures were more frequent in the most deprived group. No significant differences observed in steroid therapy use or excess alcohol consumption between the groups.

### Fragility fracture models

Patients in the most deprived areas based on the TDS had higher odds of reporting a MOF [OR 1.10 (95% CI 1.03–1.18)]. There was no significant association between hip fracture and the TDS [OR 1.14 (95% CI 0.98–1.34)]. Patients in the most deprived IMD deciles had increased odds of reporting both a MOF [OR 1.07 (95% CI 1.02–1.14)] and a hip fracture [OR 1.28 (95% CI 1.11–1.46)]. These results are presented in Table 2.

**Table 2.** Association between deprivation indices and fragility fractures

	Adjusted binary Townsend deprivation score models	Adjusted binary IMD decile models
Major osteoporotic fracture	1.10 (95% CI 1.03–1.18)	1.07 (95% CI 1.02–1.14)
Hip fractures	1.14 (95% CI 0.98–1.34)^a*^	1.28 (95% CI 1.11–1.46)

Logistic regression (reporting odds ratio (95% confidence interval)) examined odds of major osteoporotic and hip fractures by binary deprivation status. Patients in the most deprived groups (IMD deciles 1–5, Townsend >0) had 10% higher odds of major osteoporotic fractures by Townsend score and 7% by IMD. Hip fracture odds were 28% higher with IMD but showed no significant association with Townsend. Least deprived groups (IMD deciles 6–10, Townsend ≤0) were the reference. Models were adjusted for age, sex, smoking, alcohol, steroid therapy, RA, family history, BMI and bone density.

a Non-significant findings.

### Bone density models

Patients in the most deprived Townsend group also had greater odds of having osteoporosis at both the left femoral neck [1.45 (95% CI 1.33–1.59)] and lumbar spine [1.30 (95% CI 1.19–1.29)]. Similar associations were found in the IMD models, with increased odds of having osteoporosis at the left femoral neck [OR 1.34 (95% CI 1.24–1.45)] and lumbar spine *T*-scores [OR 1.20 (95% CI 1.13–1.29)]. Full results are shown in Table 3.

**Table 3.** Association between deprivation indices and osteoporosis diagnosis

	Adjusted binary Townsend deprivation score models	Adjusted binary IMD decile models
Left femoral neck *T*-score	1.45 (95% CI 1.33–1.59)	1.34 (95% CI 1.24–1.45)
Mean L1–L4 *T*-score	1.30 (95% CI 1.19–1.29)	1.20 (95% CI 1.13–1.29)

Logistic regression (reporting odds ratio (95% confidence interval)) examined odds of osteoporosis at femoral neck and lumbar spine by binary deprivation status. Patients in the most deprived groups (IMD deciles 1–5, Townsend >0) had significantly higher odds at both sites. Femoral neck osteoporosis was associated with 45% higher odds by Townsend score and 34% by IMD. Lumbar spine osteoporosis showed 30% higher odds with Townsend and 20% with IMD. Least deprived groups (IMD deciles 6–10, Townsend ≤0) were reference. Osteoporosis was defined as *T*-score ≤−2.5. Models adjusted for demographics, risk factors and fracture status.

### Body composition models

Patients in the most deprived groups had significantly higher levels of adiposity. Those classified as most deprived by the Townsend score had a 0.90% higher femoral fat percentage (95% CI 0.74–1.05) and a 0.84% higher abdominal fat percentage (95% CI 0.61–1.10) compared with those who were not deprived. In the IMD models, femoral and abdominal fat percentages were 0.60% (95% CI 0.47–0.72) and 0.67% (95% CI 0.49–0.85) higher, respectively, in the most deprived group. These results are summarized in Table 4.

**Table 4.** Association between deprivation indices and regional body fat distribution

	Adjusted binary Townsend deprivation score models	Adjusted binary IMD decile models
Left femoral fat %	*β* 0.90 (95% CI 0.74–1.05)	*β* 0.60 (95% CI 0.47–0.72)
Abdominal fat %	*β* 0.84 (95% CI 0.61–1.10)	*β* 0.67 (95% CI 0.49–0.85)

Linear regression (reporting beta coefficient (95% confidence interval)) assessed DXA-derived regional body composition by binary deprivation status. Patients in the most deprived groups (IMD deciles 1–5, Townsend >0) had significantly higher body fat percentages at both abdominal and femoral sites. The Townsend model showed increases of +0.84% abdominal and +0.90% femoral fat, while the IMD model demonstrated +0.67% and +0.60%, respectively. Beta coefficients represent percentage point increases relative to least deprived groups (IMD deciles 6–10, Townsend ≤0). Models were adjusted for demographics, lifestyle factors, steroid use, RA, family history, BMI and bone density. All results are significant.

### Supplementary analysis findings

Categorical IMD modelling showed lower odds of hip fracture in deciles 7 (OR 0.72; 95% CI: 0.53–0.97) and 8 (OR 0.71; 95% CI: 0.53–0.95) only, and lower odds of MOF in decile 10 (OR 0.81; 95% CI: 0.70–0.94) only, compared with the most deprived decile. All higher (>decile 1) IMD deciles were associated with reduced odds of osteoporosis at the lumbar spine and femoral neck, alongside progressively lower regional fat measures. Using the TDS as a spline, increasing deprivation was linked to a higher predicted probability of MOF (*P* = 0.031) and osteoporosis at both sites (*P* < 0.001), and to lower regional fat (*P* < 0.001), with no significant association for hip fracture (*P* = 0.112). Full results are presented in the Supplementary Material.

## Discussion

To our knowledge, there is limited research examining the association between deprivation and DXA-derived bone density in the UK. Moreover, no previous studies have directly compared multiple deprivation scores in this context. This is an important gap, as various deprivation scores are used in practice, and understanding how each relates to bone health could improve risk stratification and inform public health strategy.

Baseline characteristics differed significantly across IMD strata. Patients in the most deprived group (*n* = 10 659) had higher smoking prevalence, greater alcohol consumption, higher mean BMI and lower femoral neck BMD *T*-scores (−1.33 vs −1.29), all *P* < 0.001. These trends are consistent with existing evidence linking deprivation to increased smoking [16], alcohol consumption [17] and obesity [18]. Additionally, only 35.8% of referred individuals in our cohort were from the most deprived backgrounds. Although this represents a crude estimate without adjustment for the underlying population, the pattern observed across the extended study period may reflect socioeconomic inequalities in access to DXA scanning. Previous research indicates that even within publicly funded healthcare systems, patients from deprived backgrounds are less likely to attend DXA appointments [19], a finding further supported by an ecological study conducted in the UK by Griffiths *et al.* [20]. While not the primary aim of our study, these observations highlight the need for further research into socioeconomic inequities in DXA access.

A key finding of the present study is the consistency between the TDS and IMD scores across most outcomes, with the exception of hip fracture. This consistency is reassuring, given that both indices calculate area level deprivation using different methodologies. In the context of fracture estimation or broader public health strategy, either score could therefore be used. However, one limitation of the IMD is its frequent updates, which can limit temporal consistency [14]. In contrast, the TDS is typically calculated once per census cycle and used over a 10-year period. Given this stability, the TDS may be more suitable for bone health research and clinical practice, though we take note that IMD was more associated with fragility hip fractures which may be advantageous for hip fracture risk stratification. Additionally, while these measures consider area level deprivation this may be different to individual level deprivation which is a limitation of both tools.

While our results for fragility fractures were statistically significant, the effect sizes (except for hip fractures when using IMD) were generally modest. We believe this may reflect an underestimation of the true association, possibly due to the lower representation of deprived individuals in our cohort, as well as not being a true representation of the general population. As such, our findings may not fully capture the true effect size for deprivation and fracture odds. However, work from Mahmud *et al.* using the biobank cohort, examining the association between TDS and fractures resulting from simple falls, reported an odds ratio of 1.22 (95% CI 1.22–1.30) [12], which appears to be a reasonable value, though our values were slightly lower.

We also found evidence of a potential dose–response relationship between deprivation and diagnosing osteoporosis, adverse body composition, as well as between MOF and the TDS, consistent with Mahmud *et al.* [12]. However, we did not observe convincing evidence of a dose–response relationship for hip fractures. Given the low prevalence of hip fracture in our cohort (∼3%) and many levels in IMD, larger samples may be required to detect such an association. Bhimjiyani *et al.* reported higher deprivation to be associated with increased hip fracture risk, although FRAX variables were not adjusted for [21]. Therefore, it is reasonable to hypothesize that a dose–response relationship may exist, though further research in representative cohorts is needed to confirm this hypothesis.

Another key finding was the markedly higher odds of hip fracture among patients with greater deprivation, as measured by binary IMD. Given the significant morbidity and mortality associated with hip fractures [3], this represents a particularly important association. While the underlying reasons are likely multifactorial, possible explanations include reduced access to DXA scanning among deprived individuals [20] a higher prevalence of undiagnosed osteoporosis [22], and sub-optimal body composition [23] which may predispose to hip fracture. Additionally, research has shown that social deprivation as measured by the IMD 2007 was associated with 1.3 times higher incidence of hip fracture [24]. Existing literature has also shown that socially deprived patients experience higher rates of inpatient mortality following hip fracture [25]. Hence, given the substantial burden of undiagnosed osteoporosis and increased odds of hip fracture in this population, there may be value in incorporating deprivation indices into targeted screening strategies to identify at-risk communities. However, further research is needed to validate this approach.

By adjusting for the femoral neck *T*-score in our fracture analyses, we assessed whether socioeconomic deprivation influences fracture odds independent of BMD. The persistence of associations, including a 28% higher odds of hip fracture in the most deprived group, suggests that deprivation contributes to risk through mechanisms beyond bone density, such as increased falls risk [26], poorly maintained housing [27] or malnutrition [28]. This has important clinical implications, as BMD-based prediction tools may underestimate fracture risk in disadvantaged populations. However, factors such as bone density and smoking may act as mediators, and clarifying these pathways would require prospective mediation analyses beyond the scope of this study.

Our findings on bone density are consistent with those reported by Mahmud *et al.* who analysed the UK Biobank cohort, a population more representative of the public, and observed an association between socioeconomic deprivation and lower bone density (OR 1.16, 95% CI 1.13–1.19) [12]. We confirm their findings and extend the association between bone density and deprivation when measured with DXA, the gold-standard method for measuring bone density compared with heel ultrasound used in their study [29]. Moreover, while Mahmud *et al.* focused on osteopenia, our analysis demonstrates that deprivation is also associated with osteoporosis when measured with both the TDS and IMD deciles. We believe our findings, alongside previous studies, provide sufficient evidence for public health organizations on the existence of a social gradient in bone density. Future research should focus on how deprivation indices can be used to improve early identification and diagnosis of osteoporosis in affected communities.

Together with prior research, our findings suggest that socioeconomic deprivation should be considered a risk factor for poor bone health. Incorporating deprivation into fracture risk tools is challenging given global variability in indices, but local measures may still be valuable. A more effective application may be in guiding targeted screening strategies rather than direct inclusion in risk calculators. Further research is needed to validate our findings.

### Strengths and limitations

Strengths of this study include its large clinical cohort, adjustment for key confounders and the use of DXA, the gold standard for assessing bone density and regional body composition. Limitations include potential lack of generalisability given the clinical and predominantly white cohort, missing data and the use of area-based deprivation measures that may not capture individual circumstances. Temporal mismatch between deprivation indices and fracture events also raises the possibility of reverse causality, as fractures may themselves contribute to subsequent deprivation.

## Conclusions

We found that social deprivation, as measured by binary IMD, was associated with higher odds of major osteoporotic and hip fracture, as well as osteoporosis at the lumbar spine and femoral neck. Deprived patients also had greater fat mass in the abdominal and femoral regions. These associations were largely mirrored by the TDS when used as a binary variable, except for hip fracture, where the association was not statistically significant. Further research is needed to clarify the mechanisms by which deprivation affects bone health as well as evaluating the efficacy of screening approaches for osteoporosis that incorporate deprivation indices.

## Supplementary Material

keaf550_Supplementary_Data

## Data Availability

Data can be made available upon reasonable request to the corresponding author.
