# Supplementary material for: Deprivation indices and their association with fragility fractures and bone density: evidence from a large observational cohort
Source: Rheumatology (Oxford). 2025 Oct 17;65(1):keaf550. doi: 10.1093/rheumatology/keaf550 (PMC12862389; doi:10.1093/rheumatology/keaf550)
Supplement: keaf550_Supplementary_Data [file keaf550_supplementary_data.docx]

**Supplementary material**

**Major osteoporotic fracture outcome**

| **IMD Decile** | **OR (95% CI)** | **95% CI** | **P-value** |  |
| --- | --- | --- | --- | --- |
| IMD 1 (Most deprived) | 1.00 | Reference | Reference |  |
| IMD 2 | 0.93 | 0.80-1.08 | 0.322 |  |
| IMD 3 | 0.94 | 0.82-1.09 | 0.437 |  |
| IMD 4 | 0.91 | 0.79-1.06 | 0.224 |  |
| IMD 5 | 1.02 | 0.89-1.16 | 0.817 |  |
| IMD 6 | 0.92 | 0.81-1.04 | 0.186 |  |
| IMD 7 | 0.94 | 0.83-1.07 | 0.355 |  |
| IMD 8 | 0.92 | 0.82-1.04 | 0.193 |  |
| IMD 9 | 0.89 | 0.78-1.01 | 0.069 |  |
| IMD 10 (Least deprived) | 0.81 | 0.70-0.94 | 0.004 | * |

**Table S1** – Association between IMD Deciles and Major Osteoporotic Fracture. Progressive reduction in fracture risk with decreasing deprivation levels. Least deprived patients (IMD 10) had 19% lower odds versus most deprived (IMD 1, reference). Most other deciles showed non-significant risk reductions. Models adjusted for FRAX variables: age, sex, BMI, smoking, alcohol, steroid therapy, rheumatoid arthritis, family history, and bone density. OR<1.0 indicates lower risk versus most deprived group. *Significant results.


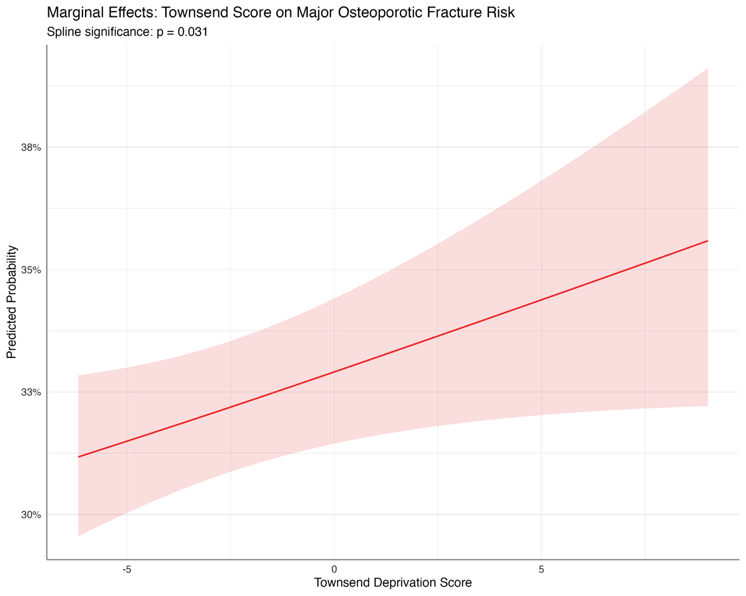


**Figure S1** – Marginal effects of Townsend Deprivation Score on Major Osteoporotic Fracture
Continuous Townsend deprivation score shows linear relationship with fracture odds. Higher deprivation scores associated with increased fracture probability. Spline analysis adjusted for FRAX variables demonstrates dose-response relationship across deprivation spectrum. Shaded area represents 95% confidence intervals.

**Hip Fracture outcome**

| **IMD Decile** | **OR (95% CI)** | **95% CI** | **P-value** |  |
| --- | --- | --- | --- | --- |
| IMD 1 (Most deprived) | 1.00 | Reference | Reference |  |
| IMD 2 | 0.89 | 0.62-1.26 | 0.499 |  |
| IMD 3 | 0.79 | 0.56-1.12 | 0.179 |  |
| IMD 4 | 0.90 | 0.64-1.27 | 0.564 |  |
| IMD 5 | 0.96 | 0.71-1.31 | 0.814 |  |
| IMD 6 | 0.78 | 0.58-1.05 | 0.100 |  |
| IMD 7 | 0.72 | 0.53-0.97 | 0.029 | * |
| IMD 8 | 0.71 | 0.53-0.95 | 0.020 | * |
| IMD 9 | 0.76 | 0.56-1.03 | 0.081 |  |
| IMD 10 (Least deprived) | 0.76 | 0.54-1.07 | 0.116 |  |

**Table S2** – Association between IMD Deciles and Hip Fracture. Substantial risk reduction in moderately affluent areas. IMD 7 and 8 showed 28–29% lower hip fracture odds versus most deprived (IMD 1, reference). Other deciles showed non-significant risk reductions. Models adjusted for FRAX variables. OR<1.0 indicates lower risk versus most deprived group. Significant results denoted by *.


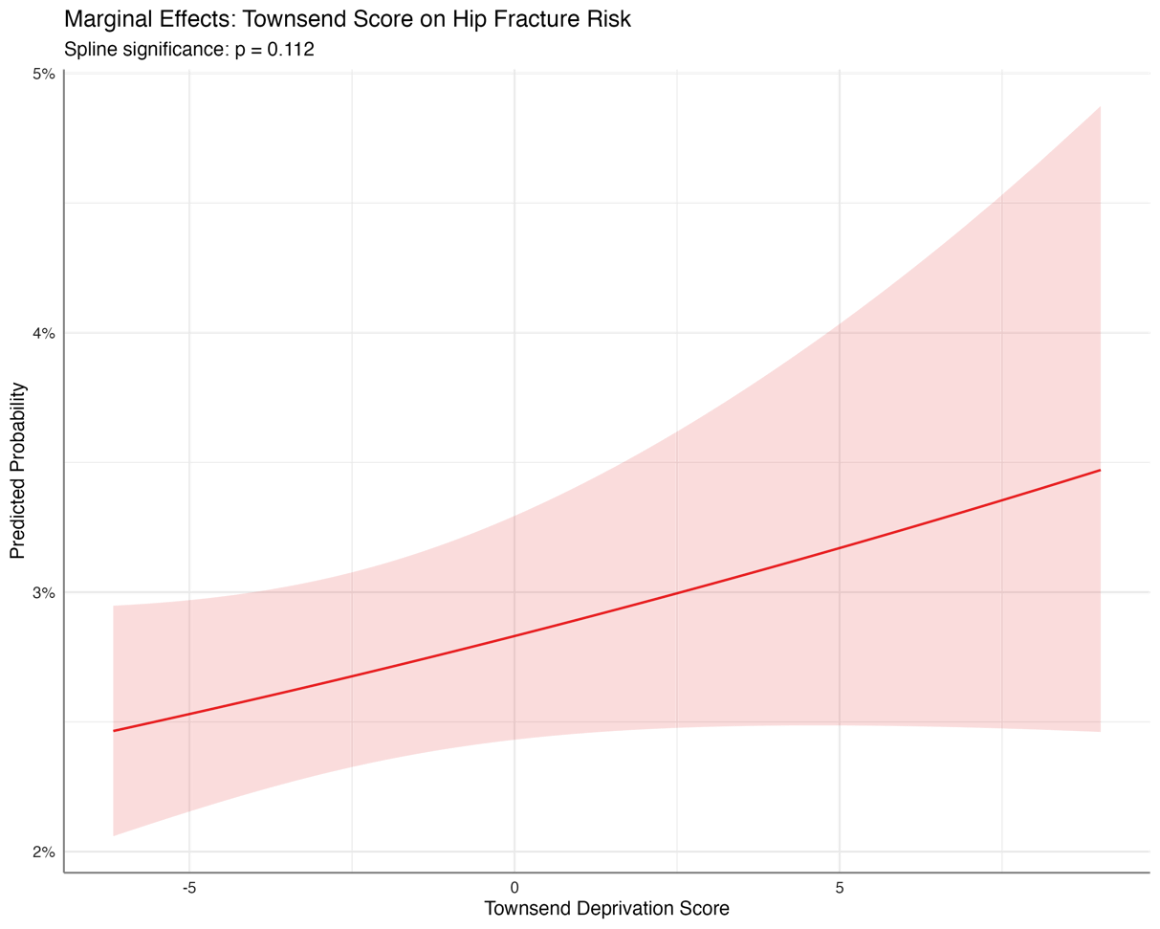


**Figure S2** – Marginal effects of Townsend Deprivation Score on Hip Fracture. Continuous Townsend score demonstrates non-linear relationship with hip fracture risk. Increasing deprivation associated with higher predicted probability of a hip fracture. Spline analysis adjusted for FRAX variables shows protective effects in moderately deprived areas. Shaded area represents 95% confidence intervals.

**Left femoral neck osteoporosis outcome**

| **IMD Decile** | **OR (95% CI)** | **95% CI** | **P-value** |  |
| --- | --- | --- | --- | --- |
| IMD 1 (Most deprived) | 1.00 | Reference | Reference |  |
| IMD 2 | 0.80 | 0.66-0.98 | 0.031 | * |
| IMD 3 | 0.77 | 0.63-0.93 | 0.007 | * |
| IMD 4 | 0.76 | 0.63-0.93 | 0.006 | * |
| IMD 5 | 0.69 | 0.57-0.82 | <0.001 | * |
| IMD 6 | 0.61 | 0.51-0.72 | <0.001 | * |
| IMD 7 | 0.64 | 0.54-0.75 | <0.001 | * |
| IMD 8 | 0.57 | 0.48-0.67 | <0.001 | * |
| IMD 9 | 0.63 | 0.53-0.74 | <0.001 | * |
| IMD 10 (Least deprived) | 0.54 | 0.45-0.66 | <0.001 | * |

**Table S3** – Association between IMD Deciles and Femoral Neck Osteoporosis. Strong protective gradient across all deprivation levels. All deciles (IMD 2–10) showed significantly lower osteoporosis odds versus most deprived (IMD 1). Greatest protection in IMD 8 (43% lower odds) and IMD 10 (46% lower odds). Models adjusted for FRAX variables. Osteoporosis defined as T-score ≤-2.5. OR<1.0 indicates lower odds. *All significant.


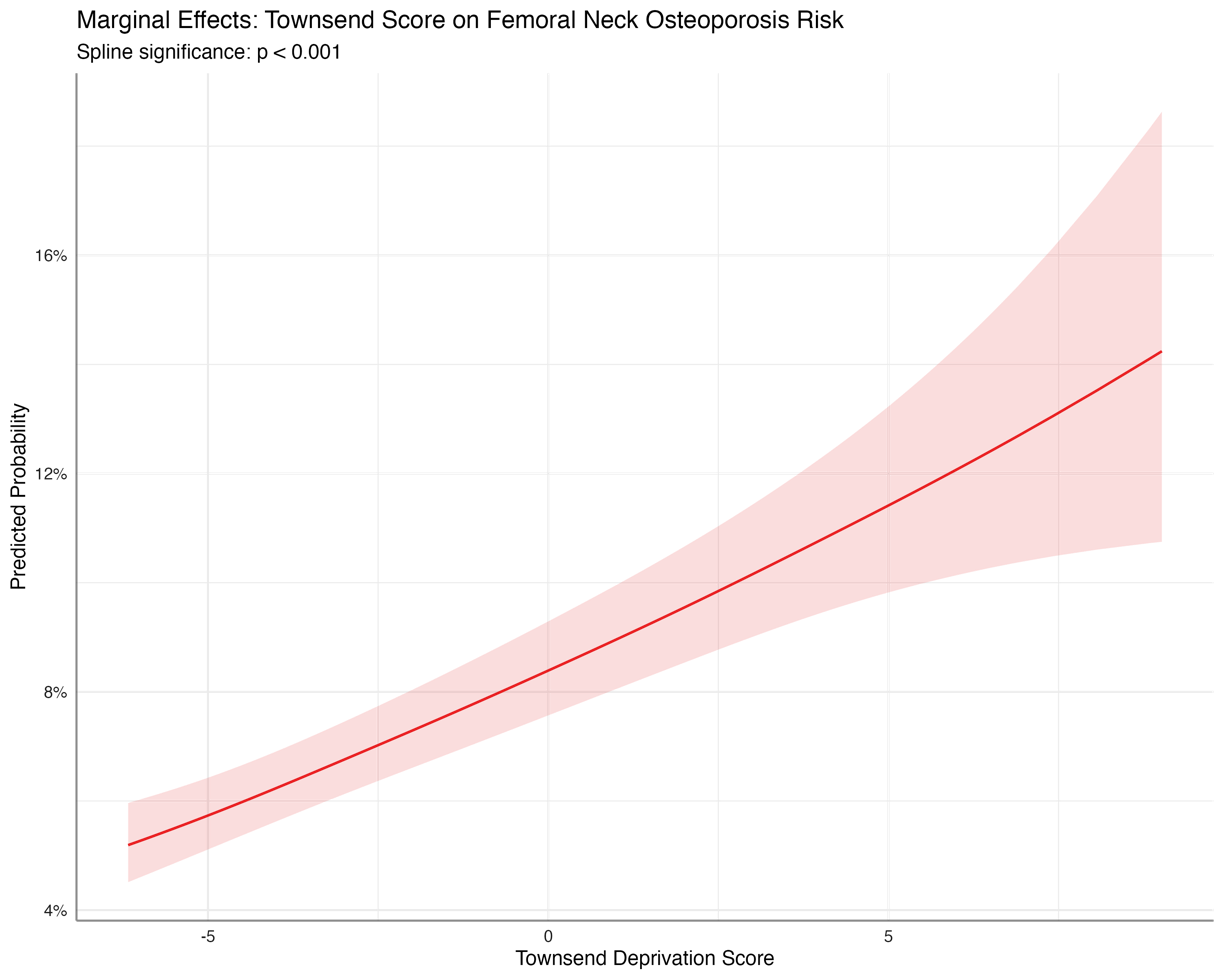


**Figure S3** – Marginal effects of Townsend Deprivation Score on Femoral Neck Osteoporosis. Continuous Townsend score shows strong linear relationship with femoral neck osteoporosis risk. Consistent increase in the predicted probability of osteoporosis with increasing deprivation. Spline analysis adjusted for FRAX variables demonstrates consistent dose-response. Shaded area represents 95% confidence intervals.

**Lumbar spine osteoporosis outcome**

| **IMD Decile** | **OR (95% CI)** | **95% CI** | **P-value** |  |
| --- | --- | --- | --- | --- |
| IMD 1 (Most deprived) | 1.00 | Reference | Reference |  |
| IMD 2 | 0.80 | 0.66-0.95 | 0.013 | * |
| IMD 3 | 0.83 | 0.70-0.99 | 0.041 | * |
| IMD 4 | 0.75 | 0.63-0.90 | 0.002 | * |
| IMD 5 | 0.73 | 0.62-0.85 | <0.001 | * |
| IMD 6 | 0.68 | 0.58-0.79 | <0.001 | * |
| IMD 7 | 0.63 | 0.55-0.74 | <0.001 | * |
| IMD 8 | 0.72 | 0.62-0.83 | <0.001 | * |
| IMD 9 | 0.63 | 0.53-0.73 | <0.001 | * |
| IMD 10 (Least deprived) | 0.71 | 0.60-0.84 | <0.001 | * |

**Table S4** – Association between IMD Deciles and Lumbar Spine Osteoporosis. Consistent protective effects across all deprivation levels when compared to decile 1 . All deciles (IMD 2–10) showed significantly lower osteoporosis odds versus most deprived (IMD 1). Risk reductions ranged 17–37%, with strongest protection in IMD 7 and 9. Models adjusted for FRAX variables. Osteoporosis defined as T-score ≤-2.5. OR<1.0 indicates lower odds. *All significant.


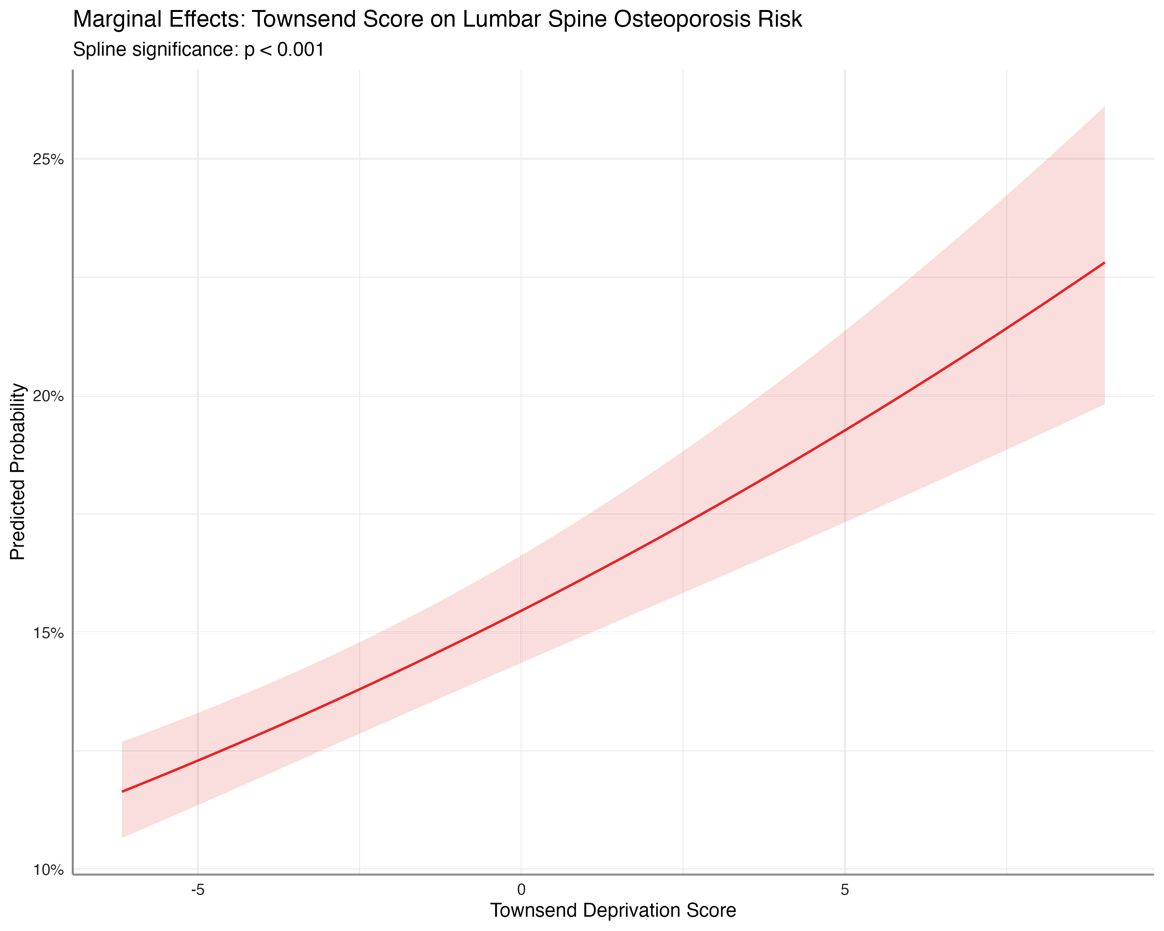


**Figure S4** – Marginal effects of Townsend Deprivation Score on Lumbar Spine Osteoporosis. Continuous Townsend score demonstrates linear relationship with lumbar spine osteoporosis risk. Consistent increase in the predicted probability of osteoporosis with increasing deprivation. Spline analysis adjusted for FRAX variables shows steady dose-response. Shaded area represents 95% confidence intervals.

**Left femoral fat percentage outcome**

| **IMD Decile** | **β (95% CI)** | **95% CI** | **P-value** |  |
| --- | --- | --- | --- | --- |
| IMD 1 (Most deprived) | 0.00 | Reference | Reference |  |
| IMD 2 | -0.180 | -0.499-0.138 | 0.267 |  |
| IMD 3 | -0.434 | -0.744--0.123 | 0.006 | * |
| IMD 4 | -0.541 | -0.853--0.229 | <0.001 | * |
| IMD 5 | -1.138 | -1.425--0.851 | <0.001 | * |
| IMD 6 | -1.156 | -1.424--0.888 | <0.001 | * |
| IMD 7 | -1.055 | -1.319--0.791 | <0.001 | * |
| IMD 8 | -1.123 | -1.384--0.861 | <0.001 | * |
| IMD 9 | -1.161 | -1.437--0.885 | <0.001 | * |
| IMD 10 (Least deprived) | -1.097 | -1.399--0.796 | <0.001 | * |

**Table S5** – Association between IMD Deciles and Left Femur Fat Percentage. Progressive reduction in fat percentage with decreasing deprivation. Less deprived deciles (IMD 3–10) had 0.43–1.16 percentage points lower femur fat versus most deprived (IMD 1). Largest reductions in IMD 5–9 (≈1.1%). Models adjusted for FRAX variables. Negative β indicates lower fat versus most deprived. *Significant results.


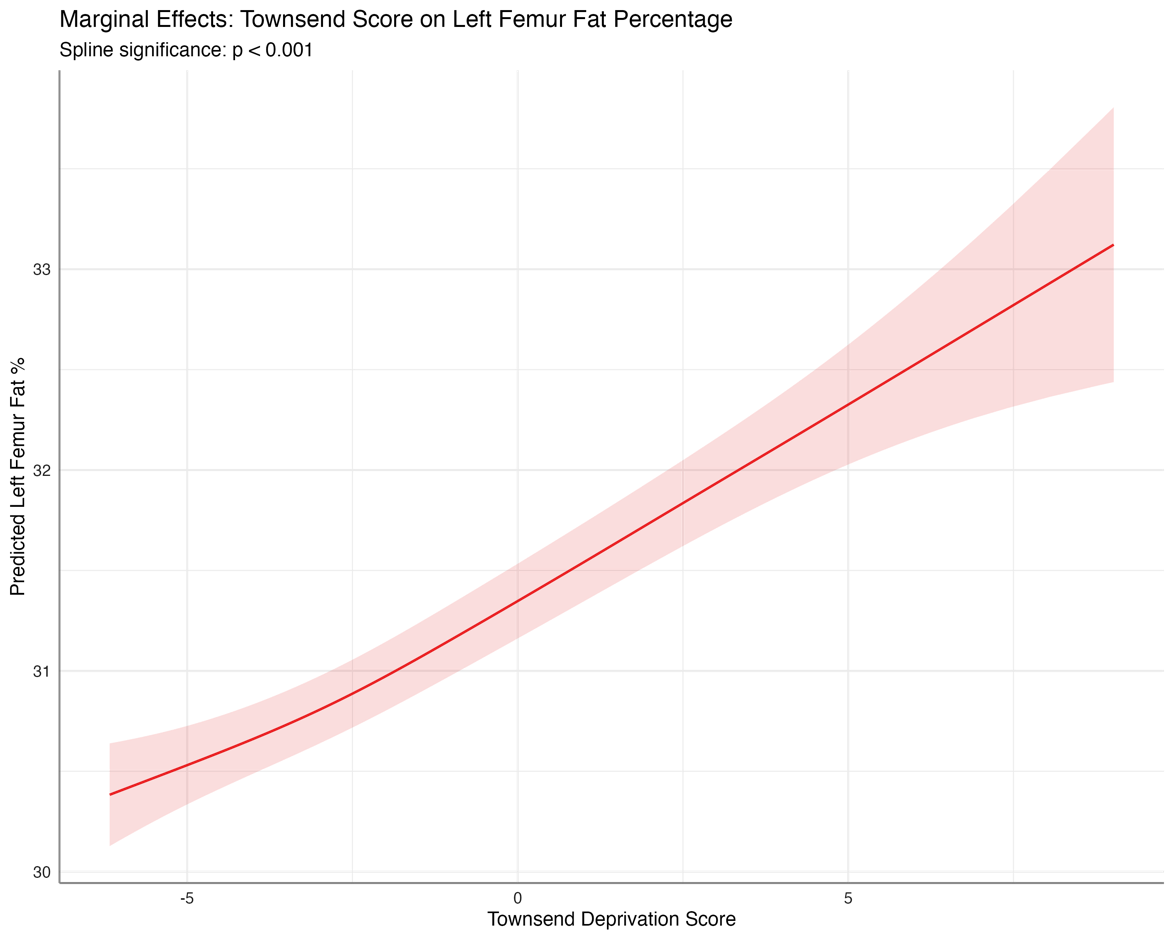


**Figure S5** – Marginal effects of Townsend Deprivation Score on Left Femoral Fat Percentage. Continuous Townsend score shows linear relationship with regional adiposity. Higher deprivation scores associated with increased femur fat percentage. Spline analysis adjusted for FRAX variables demonstrates consistent dose-response across spectrum. Shaded area represents 95% confidence intervals.

**Abdominal fat (L1 – L4 DXA region) percentage**

| **IMD Decile** | **β (95% CI)** | **95% CI** | **P-value** |  |
| --- | --- | --- | --- | --- |
| IMD 1 (Most deprived) | 0.00 | Reference | Reference |  |
| IMD 2 | -0.300 | -0.777-0.177 | 0.217 |  |
| IMD 3 | -0.432 | -0.897-0.034 | 0.069 |  |
| IMD 4 | -0.761 | -1.228--0.293 | 0.001 | * |
| IMD 5 | -0.934 | -1.364--0.504 | <0.001 | * |
| IMD 6 | -1.179 | -1.580--0.778 | <0.001 | * |
| IMD 7 | -1.059 | -1.454--0.664 | <0.001 | * |
| IMD 8 | -1.401 | -1.792--1.009 | <0.001 | * |
| IMD 9 | -1.115 | -1.528--0.701 | <0.001 | * |
| IMD 10 (Least deprived) | -1.173 | -1.624--0.722 | <0.001 | * |

**Table S6** – Association between IMD Deciles and Abdominal Fat Percentage. Substantial reduction in abdominal adiposity with decreasing deprivation. Less deprived deciles (IMD 4–10) had 0.76–1.40 percentage points lower abdominal fat versus most deprived (IMD 1). Greatest reductions in IMD 8 (1.40%) and IMD 6 (1.18%). Models adjusted for FRAX variables. Negative β indicates lower fat percentages. *Significant results.


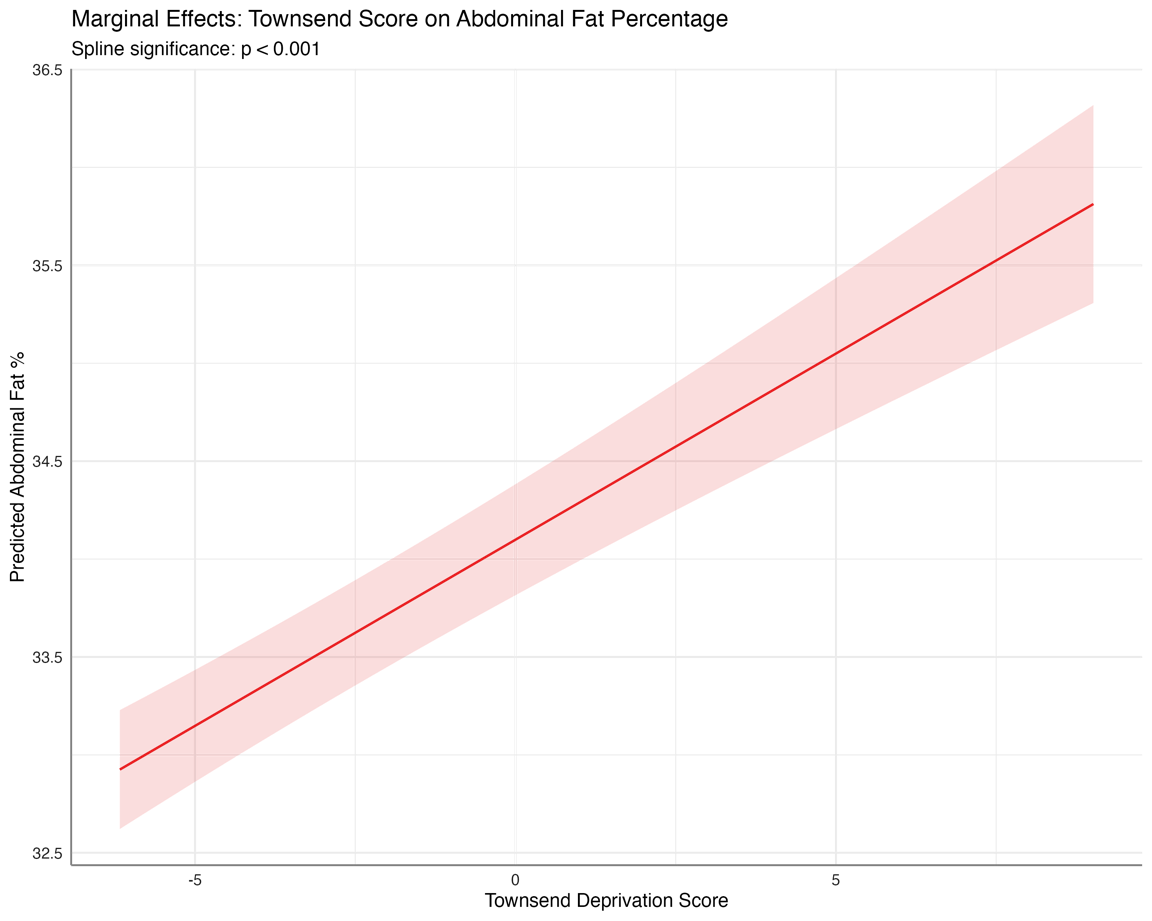


**Figure S6** – Marginal effects of Townsend Deprivation Score on Abdominal Fat Percentage. Continuous Townsend score demonstrates linear relationship with central adiposity. Progressive increase in abdominal fat percentage with higher deprivation scores. Spline analysis adjusted for FRAX variables shows consistent dose-response pattern across full deprivation range. Shaded area represents 95% confidence intervals
